# The Potential Roles of a Laminin Receptor in Adhesion and Apoptosis of Cells of the Marine Bivalve *Meretrix meretrix*

**DOI:** 10.1371/journal.pone.0047104

**Published:** 2012-10-09

**Authors:** Yanan You, Pin Huan, Xiaomei Wang, Baozhong Liu

**Affiliations:** 1 Key Laboratory of Experimental Marine Biology, Institute of Oceanology, Chinese Academy of Sciences, Qingdao, China; 2 Graduate School of the Chinese Academy of Science, Beijing, China; 3 Research Center of Resources and Eco-Environment, Chinese Academy of Fishery Sciences, Beijing, China; University of Edinburgh, United Kingdom

## Abstract

**Background:**

The laminin receptors (LRs) play important roles in cell adhesion to the extracellular matrix, certain cell-cell adhesions, and the activation of many intracellular signaling pathways. Studies of LRs have primarily focused on mammals, while few studies of LRs in marine invertebrates have been reported. The functions of LRs in marine bivalve species are still unclear.

**Methodology/Principal Findings:**

In this study, we cloned and sequenced an LR gene, MmeLR, from the clam *Meretrix meretrix*. The MmeLR mRNA and protein detected by realtime PCR and western blots were primarily distributed in muscle tissues. Far-western analysis showed a specific interaction between recombinant MmeLR and the LR ligand laminin. The results of the binding assay suggested a role of LR in cell adhesion and apoptosis in cultured primary cells of mantle tissues from *M. meretrix*. The Bcl-2 mRNA expression level in primary cells cultured in matrigel (mainly laminin) coated plates was significantly higher than in cells cultured in non-coated plates at 48 h of culture, while the p53 mRNA expression pattern was inversely related to that of bcl-2, suggesting that MmeLR is involved in p53-dependent apoptosis, and the binding between MmeLR and laminin inhibits apoptosis during primary cell culture.

**Conclusions:**

Our results suggest that MmeLR may be involved in cell adhesion and apoptosis. This study may increase the understanding of the role of laminin receptor in cell adhesion and apoptosis and help to improve the culture of primary cells of marine invertebrates.

## Introduction

Laminins are extracellular matrix proteins found in the basement membrane of cells and are involved in many important biological events, such as cell growth, cell differentiation and migration, cell adhesion to the extracellular matrix, and cell-cell adhesions [1]–[3]. Laminins perform their physiological functions by binding to their receptors. Laminin receptors (LRs) are transmembrane glycoproteins. In addition to mediating cell adhesion, LRs make transmembrane connections to the cytoskeleton and activate many intracellular signaling pathways [4]. The LRs and the pathways they regulate contribute to cell differentiation, migration, and the generation and development of organs [2], [5]–[7].

There are at least three types of laminin binding domains on the LRs [8]. The LRs can be divided into two groups: integrin and non-integrin receptors, according to the mechanism used for binding to laminins [9]. The 67 kDa LR is a non-integrin receptor [10], and a highly conserved 37 kDa protein is the precursor of the 67 kDa LR [11]. Terranova et al (1983) first found a 67 kDa LR in human mastadenoma cells [12]. Subsequently, multiple signal transduction pathways mediated by the 67 kDa LR with laminin were reported, involving various components such as mitogen activated protein kinases (MAPK), dual specificity phosphatases (DUSP), intracellular calcium, and calmodulin kinase II (CAMK II) [13]–[15]. Recently, researchers found that many pathways regulating the origin, differentiation, and dissemination of cancer cells were mediated by the 67 kDa LR [16].

Studies of LRs have focused primarily on mammals, for instance, the embryo development and organogenesis of mice [17], [18], and the motility, differentiation, invasion, adhesion, proliferation and apoptosis of human cancer cells [13], [19], [20]. However, few studies of LRs in marine invertebrates have been reported. In marine bivalves, the only report was that of Fu et al (2008), who cloned and characterized an LR precursor gene in the pearl oyster *Pinctada fucata* [21]. Consequently, the functions of LRs in marine bivalve species are still largely unknown.

Due to their functions in cell adhesion and apoptosis, LRs may exhibit physiological activities in marine invertebrates similar to those in vertebrates. For decades, many attempts have been made to establish cell lines of marine invertebrates, and many methodologies have been developed; however, a permanent cell line from marine invertebrates has not been obtained [22]. Fortunately, primary cell cultures of some marine invertebrates are available. Nevertheless, most of the present cultured primary cells are short-lived because many obstacles exist, such as poor adherence [22]. The functions of LRs in cell adhesion are reminiscent of the potential applications of these molecules in facilitating cell adhesion during cell culture, and thus LRs might contribute to establishing a permanent cell line. Besides, many recent studies have indicated that apoptosis plays an important role in molluscan development and immune defense [23], [24]. Thus, the elucidation of the functions of LRs in cell adhesion and apoptosis is crucial for the study of clam biology.

The clam *Meretrix meretrix* is a representative species of marine bivalve and an important commercial bivalve in China [25]. In the present study, we cloned and characterized a 37 kDa laminin receptor precursor gene from *M. meretrix* and named it MmeLR. Its tissue expression characteristics were examined on both the transcriptional and translational levels. Binding assays were conducted to investigate the role of MmeLR in cell adhesion and apoptosis in the primary cells of mantle tissues from *M. meretrix*. This study may lead to an improved understanding of the biological functions of laminin receptors in marine invertebrate species.

## Results

### MmeLR cDNA Sequence

**Figure 1:**
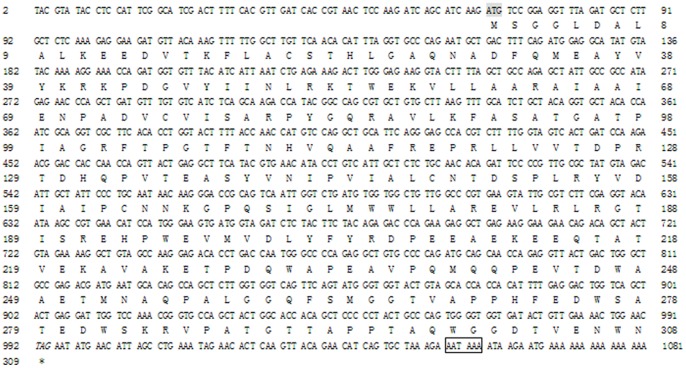
The cDNA sequence and the deduced amino acid sequence of MmeLR. Numbering of the nucleotide and amino acid sequences is shown on the left and right, respectively. The initiation codon is shadowed and the poly-adenylation signal is bordered.

A 1081 bp cDNA containing a 927 bp open reading frame (ORF) was identified by 5′ and 3′ RACEs (Fig. 1). Analysis of the 3′-untranslated region (3′-UTR) revealed a canonical poly-adenylation signal (AATAAA) and a poly (A) tail. The ORF encoded a polypeptide of 308 amino acid residues with a theoretical molecular mass of 34.039 kDa and a pI of 5.02. Signal P prediction indicated no signal peptide in MmeLR. The MmeLR cDNA and the deduced amino acid sequence have been deposited in GenBank under the accession number JQ973062.

### Homology and Phylogenetic Analysis of MmeLR

**Figure 2:**
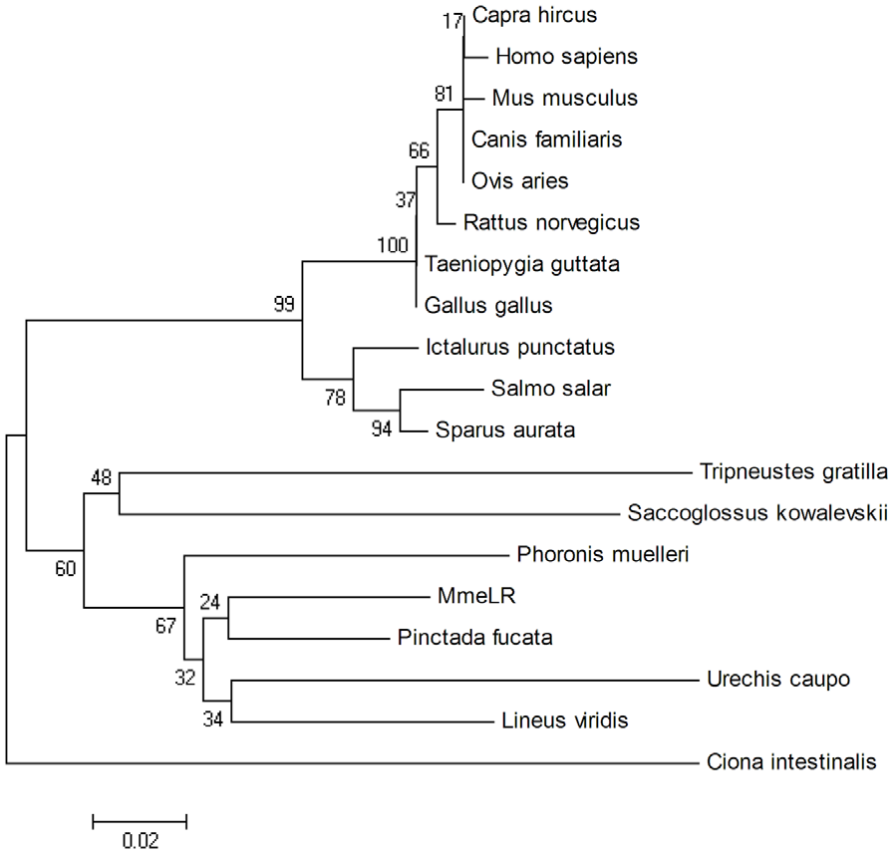
Phylogenetic analysis of MmeLR. The tree was constructed using the neighbor-joining method. The bootstrap confidence was calculated from 1000 replications.

BlastX searches in the NCBI database revealed that the deduced amino acid sequence of MmeLR shared 78% sequence identity with the 67 kDa laminin receptor precursor from the pearl oyster *P. fucata* (Accession No. ABO10190.1). A phylogenetic tree was generated using the information from LRs of other species (Table 1), and it showed that MmeLR was placed basally in a monophyletic clade formed by five marine species (*Urechis caupo, Lineus viridis, Phoronis muelleri, Saccoglossus kowalevskii,* and *Tripneustes gratilla*), close to the 67 kDa laminin receptor precursor of the pearl oyster *P. fucata* (Fig. 2).

**Table 1:** Information about the sequences used in the phylogenetic analysis.

Species	Name of gene	Accession number
*Pinctada fucata*	67 kD laminin receptor precursor	ABO10190.1
*Lineus viridis*	ribosomal protein rpsA	ABZ04275.1
*Sparus aurata*	40S ribosomal protein Sa-like protein	AAT44424.1
*Saccoglossus kowalevskii*	ribosomal protein SA-like	XP_002742035.1
*Ictalurus punctatus*	40S ribosomal protein SA	NP_001187066.1
*Taeniopygia guttata*	putative laminin receptor 1	ACH43847.1
*Gallus gallus*	40S ribosomal protein SA	NP_001007824.1
*Homo sapiens*	40S ribosomal protein SA	NP_002286.2
*Urechis caupo*	34/67 kD laminin binding protein	AAA90978.1
*Ovis aries*	laminin receptor 1	ABR57322.1
*Mus musculus*	mCG2650	EDL11947.1
*Canis familiaris*	40S ribosomal protein SA-like isoform 1	XP_533909.1
*Tripneustes gratilla*	34/67 kD laminin binding protein	AAA90977.1
*Rattus norvegicus*	laminin receptor	ACJ13448.1
*Salmo salar*	40S ribosomal protein SA	NP_001134397.1
*Ciona intestinalis*	similar to 67 kD laminin receptor	XP_002126356.1
*Phoronis muelleri*	putative 40S ribosomal protein RPSA	ACD65134.1
*Capra hircus*	laminin receptor 1	ADI56590.1

### Immunofluorescence Analysis of Laminin

**Figure 3:**
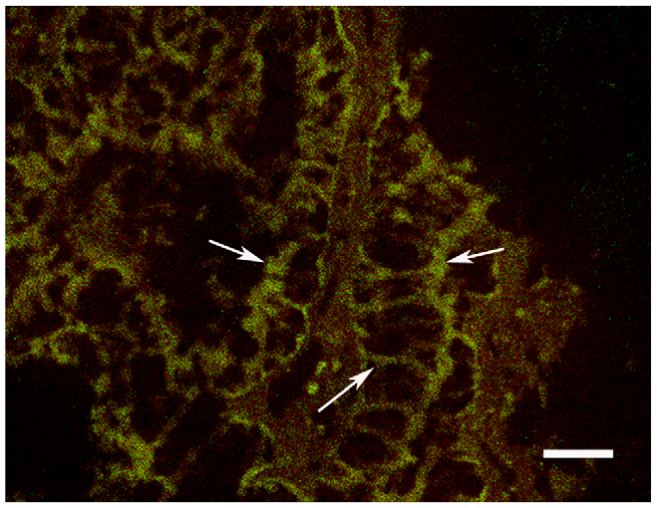
Immunofluorescence analysis of laminin. White arrows show the location of laminin detected by FITC (green) signals. Red signals indicate the outline of the mantle tissue slices. The laminin is primarily localized in the ECM of the clam, *M. meretrix*.

In the immunofluorescence test, the location of laminin was detected by FITC (green) signals (Fig. 3). Red signals indicated the outline of the mantle tissue slices. The negative control slides incubated in the absence of primary antibodies showed no green immunostaining (data not shown). The results showed that laminin is primarily localized in the extracellular matrix (ECM) of the clam *M. meretrix*.

### Interaction between rMmeLR and Laminin

**Figure 4:**
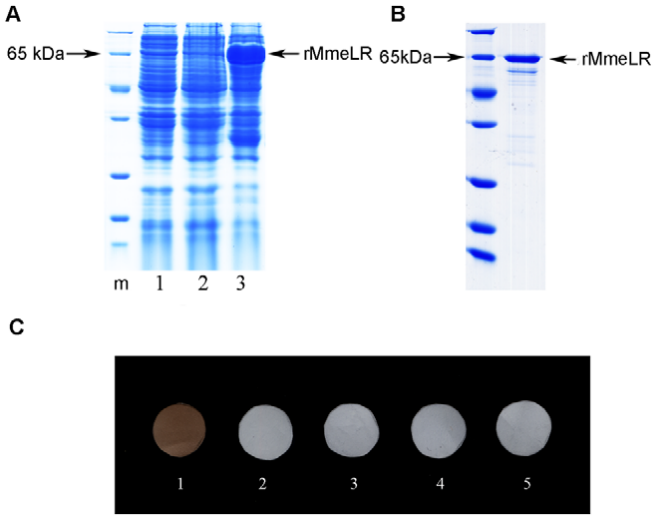
The production of recombinant protein MmeLR and far-western analysis of rMmeLR and laminin. (A) A distinct band of 65 kDa was observed, which was consistent with the mass of the 37 kDa laminin receptor precursor protein plus the GST tag (28 kDa). m: marker; Lane 1: the negative control bacteria; Lane 2: bacteria with the pGEX-MmeLR recombinant plasmid without IPTG induction; Lane 3: bacteria with the pGEX-MmeLR recombinant plasmid with IPTG induction for 4 h at 37°C. (B) The rMmeLR protein was purified by affinity chromatography and then detected by SDS-PAGE and CBB-R250 staining. (C) Far-western analysis showed specific binding between rMmeLR and laminin. 1: Laminin on the membrane and incubation with rMmeLR; 2: rCaspase on the membrane and incubation with rMmeLR; 3: Laminin on the membrane and incubation with GST protein; 4: rCaspase on the membrane and incubation with GST protein; 5: blank control (no protein on the membrane and incubation with rMmeLR).

A recombinant MmeLR (rMmeLR) protein was obtained by prokaryotic expression. When SDS-PAGE was performed to check the expression of rMmeLR, a distinct band with a molecular weight of approximately 65 kDa was observed by Coomassie brilliant blue R250 staining (Fig. 4A). The size is consistent with the molecular mass of the precursor protein of the laminin receptor predicted from the cDNA sequence (37 kDa) plus the GST tag (28 kDa). The rMmeLR protein was purified by affinity chromatography, and a single band was detected by SDS-PAGE (Fig. 4B).

**Figure 5:**
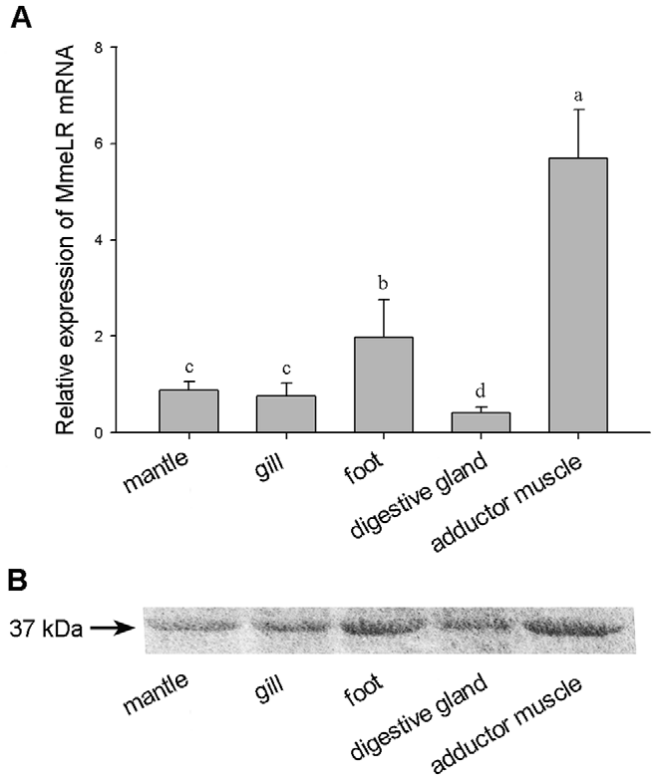
The tissue-specific expression profile of MmeLR. (A) The mRNA expression levels in the five tissues from *M. meretrix.* The MmeLR mRNA expression levels were higher in the adductor muscle and foot than in the other three tissues, and the lowest level was in the digestive gland. Different letters indicate significant differences between different tissues at *P*<0.05. (B) The MmeLR protein expression was analyzed by western blot. The results showed the same expression profile for the protein as for the mRNA in the five tissues.

Using rabbit anti-rMmeLR polyclonal antibodies, we conducted far-western assays to investigate the binding ability of rMmeLR to its ligand. The results showed rMmeLR could bind to laminin specifically (Fig. 4C, 1), whereas rMmeLR did not bind to the unrelated protein rCaspase (Fig. 4C, 2). The membranes containing laminin and rCaspase incubated with GST protein showed no positive signal (Fig. 4C, 3, 4), which eliminated the potential influence of the GST tag of rMmeLR. The control (membrane containing no protein) also had no positive signal (Fig. 4C, 5).

### The Tissue-specificity of MmeLR Expression

Realtime PCR assay was used to detect MmeLR mRNA expression in foot, gill, digestive gland, adductor muscle, and mantle tissue. Only one peak was observed in melting curve analyses for each pair of primers, which ensured the specificity of the reactions. The mRNA levels were significantly higher in the adductor muscle and foot than in the other three tissues (Fig. 5A, *P*<0.05). The MmeLR protein expression was analyzed by western blot, and the result showed that the protein levels were highest in the adductor muscle and lowest in the digestive gland, which was similar to the expression profile of the mRNA (Fig. 5B).

### The Expression Pattern of MmeLR in Primary Cells

**Figure 6:**
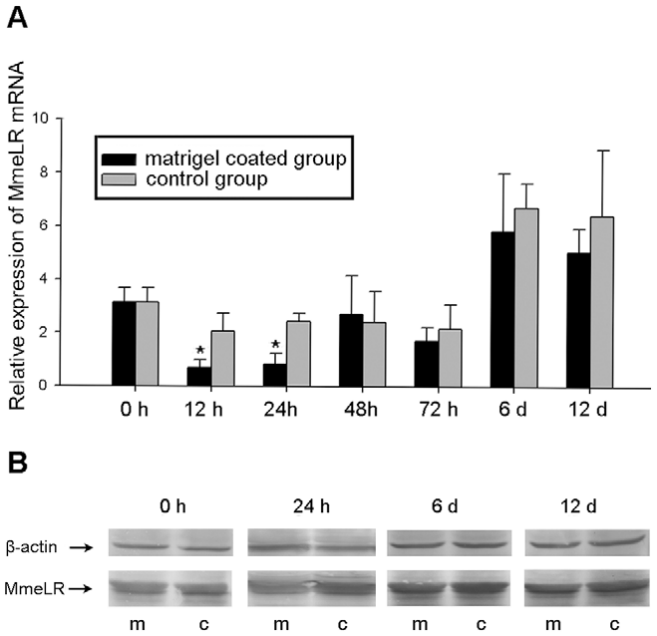
The temporal expression pattern of MmeLR in the primary cells from the binding assay. (A) Cells of the matrigel-coated group and the control group were harvested at 0 h, 12 h, 24 h, 48 h, 72 h, 6 days and 12 days. At 6 days and 12 days, the MmeLR mRNA expression levels of both groups were approximately 2 – 2.5-fold higher than at earlier time points (*P*<0.05). At 12 h and 24 h, the MmeLR mRNA expression levels of the control group were approximately 2.5-fold higher than that of the matrigel-coated group, and the difference was significant. *indicates significant difference (*P*<0.05). (B) Cells of the two groups were harvested at 0 h, 24 h, 6 days and 12 days for western blots. The β-actin protein (42 kDa) was used as the endogenous reference. Lane m: Cells of the matrigel-coated group. Lane c: Cells of the control group. At 24 h, cells of the matrigel-coated group expressed less MmeLR than the control group. At 6 days and 12 days, in both groups, MmeLR was expressed at higher levels than at 24 h.

Realtime PCR was used to analyze the expression pattern of MmeLR mRNA in cells in different culture conditions at seven time points during 12 days. At 12 h and 24 h, there was a significant difference between the cells on matrigel-coated plates and the control cells on uncoated plates (Fig. 6A). The MmeLR mRNA expression level of the control group was approximately 2.5-fold higher than that of the matrigel-coated group at 12 h and 24 h. The results also showed that at 6 days and 12 days, the MmeLR mRNA expression levels in the two groups were approximately 2–2.5-fold of those of the first three days, differences that were statistically significant (*P*<0.05).

Western blots showed that the MmeLR protein expression pattern was the same as the mRNA expression pattern. Cells on the matrigel-coated plates expressed less MmeLR protein than the control cells at 24 h, whereas there was no significant difference between the two groups at 6 days and 12 days. The expression levels of the MmeLR protein at 6 days and 12 days were greater than at the earlier time points in both sets of cells (Fig. 6B).

### Effects of MmeLR on the Apoptosis of Primary Cells

**Figure 7:**
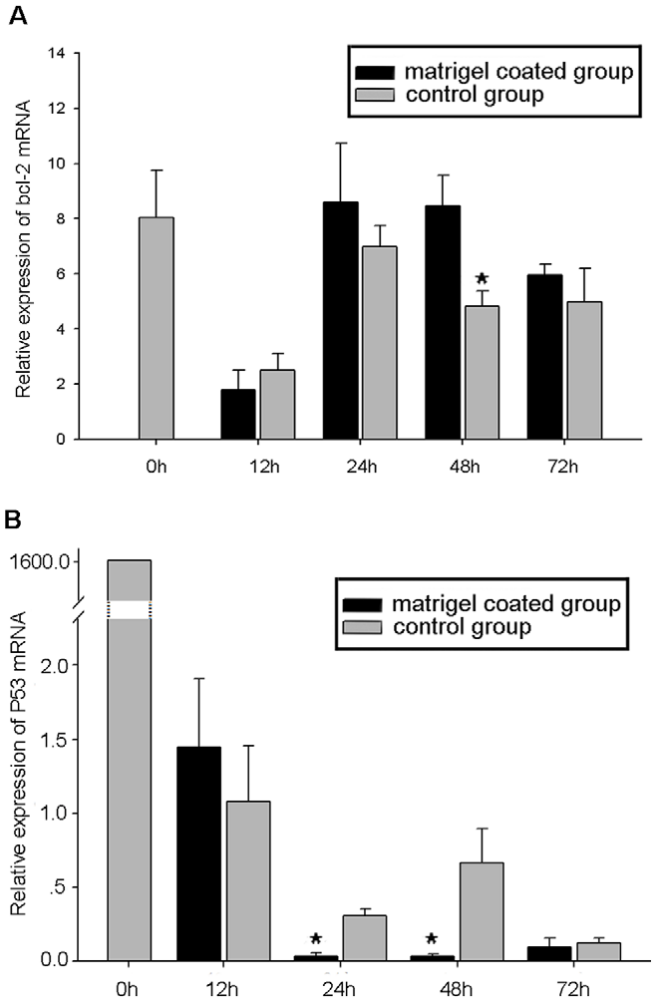
The effect of LRs on apoptosis of primary cells. Bcl-2 and p53 mRNA expression levels were used to estimate apoptosis. (A) At 48 h, the bcl-2 mRNA expression level of the matrigel-coated group was significantly higher than that of the control group (*P*<0.05), while there were no significant differences between the two groups at other time points. (B) At 24 h and 48 h, the p53 mRNA expression levels of the matrigel-coated group were significantly lower than those of the control group (*P*<0.05). At 72 h, the cells of the control group expressed less p53 mRNA than during the first two days, and there was no obvious difference between the two groups. *indicates significant differences (*P<*0.05) in the mRNA expression levels of bcl-2 and p53 in the two groups.

Bcl-2 and p53 were used to indicate the apoptosis condition of cultured cells. At 48 h, the bcl-2 mRNA expression level was significantly higher in the matrigel-coated group than in the control group (*P*<0.05), and there were no differences between the two groups at the other time points (Fig. 7A). The p53 mRNA expression pattern was inversely related to that of bcl-2. At 24 h and 48 h, the p53 mRNA expression levels of the matrigel-coated group were significantly lower than those of the control group (*P*<0.05). There were no obvious differences between the p53 expression levels of the two groups at 72 h (Fig. 7B).

**Figure 8:**
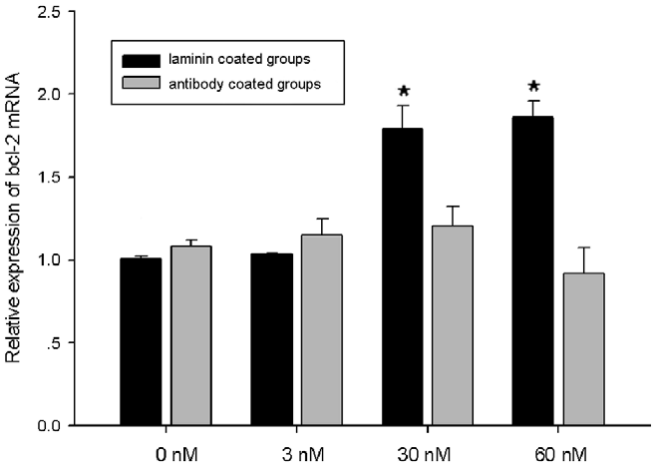
The effects of laminin and the laminin antibody on apoptosis of the primary cells. The bcl-2 mRNA expression level was used to estimate the cellular apoptosis condition. At 48 h, the bcl-2 mRNA expression levels of the cells in the 30 nM and 60 nM laminin coated groups were significantly higher than in the 0 nM and 3 nM groups (*P*<0.05, black columns, *). The bcl-2 expression levels of the four laminin antibody coated groups (gray columns) did not differ significantly from those of the blank control group (no laminin nor its antibody coated), indicating that the effect of laminin on apoptosis was blocked by the antibody. *indicates a significant difference (*P<*0.05) in bcl-2 mRNA expression between the groups at 48 h.

Experiments with laminin and laminin antibody were conducted to confirm the roles of laminin in apoptosis of the primary cells. At 48 h, the bcl-2 mRNA expression levels of the cells on plates coated with 30 nM and 60 nM laminin were significantly higher than the expression levels of the cells on plates without laminin (0 nM) or in plates with 3 nM laminin, indicating that laminin ameliorated apoptosis. On plates coated with laminin and saturating amounts of laminin antibody, the functions of the laminin were blocked, and cells in the four laminin antibody-coated groups expressed bcl-2 mRNA with no significant differences from the blank control group (no laminin nor its antibody coated) (Fig. 8).

## Discussion

The 67 kDa laminin receptor is a non-integrin cell-surface receptor with high affinity for laminins [9]. In the present study, a gene for a 37 kDa laminin receptor precursor was cloned from the marine bivalve *M. meretrix* and characterized. Both the mRNA and the protein of MmeLR are expressed in various tissues and most abundantly in the muscle tissues. The binding of MmeLR to its ligand laminin suggested a role of MmeLR in cell adhesion and apoptosis.

The gene coding for the 67 kDa laminin receptor is highly conserved, and the protein contains the same amino acid residues in different species [11], [26]. The MmeLR gene encoded 308 amino acid residues, and phylogenetic analysis revealed that MmeLR was homologous to the 67 kDa laminin receptor from the pearl oyster *P. fucata.* A 37 kDa protein is the precursor of the 67 kDa laminin receptor, but the exact mechanism by which the mature protein is formed is still not clear [13]. Rao et al (1989) and Buto et al (1998) suggested that the acylated 37 kDa precursor forms the mature 67 kDa laminin receptor by homo- or heterodimerization through non-covalent bonding [11], [27]. In addition, the cDNA encoding the 37 kDa laminin receptor precursor also encodes the ribosomal protein p40, suggesting that the protein participates in the translational mechanism [28].

In the pearl oyster *P. fucata*, LR is especially highly expressed in the mantle [21], which is mainly composed of muscle tissues. We found that the MmeLR mRNA was also expressed abundantly in the muscle tissues; however, it was higher in the adductor muscle and foot tissues. The same profile was observed for the MmeLR protein expression. Because MmeLR was highly expressed in the muscle tissues, it might be functional in muscle, playing a role in movement and signal transduction [29], [30]. In addition, the relatively high expression level in mantle tissue suggested that MmeLR may function in cell proliferation and cell connection with the matrix [21].

As a receptor, the laminin receptor functions in combination with its ligands. Far-western analysis showed that there was a specific interaction between rMmeLR and laminin. In the present study, primary cells of mantle tissues were cultured to analyze the cellular response to the binding of MmeLR to laminin. At 12 h and 24 h, cells cultured in matrigel-free plates (control group) expressed more MmeLR mRNA than those cultured in matrigel-coated plates. Based on the negative feedback regulation mechanism existing in the organisms [31], we presume that MmeLR was expressed at lower levels than usual when the cells were oversaturated with laminin, whereas more MmeLR would be transcribed to compete for limited laminin. On the other hand, in addition to the 67 kDa laminin receptor, laminin has other receptors on the cell surface, such as the integrins [10]. In many cases, integrins and the 67 kDa laminin receptor act together in transducing laminin effects. Therefore, based on the negative feedback regulation, when cells are oversaturated with laminin, the integrins may be reduced, which may be accompanied by a proportional decrease in the cell surface expression of the 67 kDa laminin receptor [9].

At 6 days and 12 days, the laminin added to the culture plates was basically exhausted; therefore, the MmeLR mRNA expression levels were higher than in the first two days. Recently, the 67 kDa laminin receptor was found to have other ligands in addition to laminin. The cell-surface EGCG receptor is an example [32]. EGCG participates in many signal transduction pathways involved in cancer prevention, anti-oxidative stress and anti-inflammatory activities [33]. In the present research, the higher MmeLR expression levels of the cells cultured for 6 days and 12 days would most likely be due to the relatively insufficient nutrients in the culture compared with the first two days. To activate the cell self-protecting mechanism, more MmeLR was produced to interact with extracellular ligands, such as anti-oxidants.

Our research also suggested the potential function of MmeLR in the apoptosis of the primary cells from *M. meretrix.* Bcl-2 and p53 were used to estimate the apoptosis condition [34], [35]. At 48 h after culturing, the bcl-2 mRNA expression level in the matrigel-coated group was significantly higher than in the control group, which may indicate that apoptosis of the cells in the matrigel-coated group was lagging behind that of the control group. At 0 h, cells expressed more p53 mRNA than at the other time points, most likely because cells had been recently dissociated from the tissue fragments, a procedure that stimulates complex mechanisms, and p53 mRNA expression was up-regulated. At 24 h and 48 h, the p53 mRNA expression level of the matrigel-coated group was significantly lower than that of the control group, indicating that matrigel ameliorated the p53-dependent apoptosis. It should be noted that at 48 h the p53 mRNA expression level was opposite that of bcl-2. Some research supports the theory of an inverse relationship between the expression of p53 and bcl-2, possibly because bcl-2 functions as a transcription regulator of p53 [36], [37]. Kim et al (1999) suggested that laminin-1-adherent cells showed increased proliferative activity and reduced apoptosis compared with the laminin-1-non-adherent cells [38]. The 67 kDa laminin receptor mediates cell attachment to laminin [4], and the adherence between cells and plates would greatly promote the growth of primary cells [39]. Furthermore, Rinkevich (2005) reported that when cells were dissociated from many different marine invertebrates, they stopped dividing in vitro within 24–72 h and became quiescent [9], [40]. Accordingly, in this study, we found that at 72 h there was no significant difference between the bcl-2 and p53 mRNA expression of the matrigel-coated group and the control group, most likely because the cells became quiescent.

At 48 h, both bcl-2 and p53 mRNA expression showed significant differences between the matrigel-coated group and the control groups. Thus, to determine whether laminin plays the key role in cell apoptosis, we used different concentrations of laminin to coat the culture clusters. Our results showed that at 48 h, cells in the high laminin concentrations expressed significantly more bcl-2 mRNA than cells in the lower concentrations, indicating that laminin inhibited apoptosis of the primary cells [34]. Furthermore, when the laminin antibody was used to block laminin, there were no significant differences in the bcl-2 mRNA expression levels in any of the groups. These results confirmed our hypothesis that MmLR inhibited the apoptosis of the *M. meretrix* primary cells by interacting with laminin.

In summary, we cloned and characterized an LR gene from *M. meretrix*, a gene that probably functions in cell adhesion and apoptosis. Further studies of its functions will allow us to gain more insight about the roles that LR plays in bivalves and may promote the establishment of cell lines of marine invertebrates or facilitate the culture of primary cells. On the basis of this work, we anticipate more discoveries about LR and its functions in marine bivalves.

## Materials and Methods

### Sample Collection, RNA Extraction and cDNA Synthesis

The *M. meretrix* clams were obtained from an aquatic market in Qingdao, China, and acclimatized for one week in our laboratory (25°C, 30‰ salinity and under continuous aeration).

Samples were collected and quickly frozen in liquid nitrogen. Total RNA was extracted by Unizol Total RNA Isolation Reagent (UC, Shanghai, China) from samples of mantle, gill, digestive gland, adductor muscle, and foot according to the manufacturer’s protocol. The quantity and quality of the RNA was assessed by OD_260/280_ and electrophoresis in 1.5% agarose gels. Total RNA was digested by DNase (0.06 U/µl) for 30 min at 37°C, and then cDNA was synthesized from 5 µg of total RNA by M-MLV reverse transcriptase (Promega, USA) following the manufacturer’s protocol with an oligo (dT) primer AOLP and BDA oligos (Table 2).

**Table 2:** Nucleotide sequences of primers used for MmeLR cloning and expression analysis.

Name	Sequence (5'-3')
lr-f	ATGTGGTGGCTGTTGGC
lr-r	CCTTCTCAGCCTCTTCTGG
Ap	GGCCACGCGTCGACTAGTAC
T3	ATTAACCCTCACTAAAGGGAA
w-act-f	TTGTCTGGTGGTTCAACTATG
w-act-r	TCCACATCTGCTGGAAGGTG
AOLP	GGCCACGCGTCGACTAGTAC(T)_16_(A/C/G)
BDA oligo	AAGCAGTGGTATCAACGCAGAGTACGCGGG
rtLR-f	ATCGCAGGTCGCTTCACAC
rtLR-r	GACTGCGGTCCCTTGTTATT
LRBamH I-F	AAAGGATCCGGCACCATGTCCGGAGGTTTAGATGCT
LREcoR I-R	TTTGAATTCGTTCCAGTTTTCAACAGTATCAC
bcl-2f	GTGGAGAACCGATGCTTGATAGA
bcl-2r	CACCGTAAGCGTAACAATGATGT
p53f	TGACCAGGAGACAGCAGC
p53r	GTTCATCACCCTTCTTTATC

### Cloning of Full-length MmeLR cDNA and Sequence Analysis

Specific primers (lr-f, lr-r) (Table 2) were designed according to the EST sequence (Accession No. HO206523) from the constructed cDNA library, which showed the highest identity to laminin receptors. The 5′ end of the MmeLR cDNA was amplified with lr-r and the vector primer T3. Lr-f was designed according to the amplified sequence. Rapid amplification of cDNA ends (RACE) was employed to obtain the 3′ end of the LR cDNA using lr-f and an adapter primer, AP, according to the instructions for the SMART RACE cDNA Amplification Kit (Clontech, USA). PCR products of the expected sizes were excised and extracted with gel extraction kits (Promega, USA).

The MmeLR amino acid sequence was deduced from the cDNA sequence using the software BioEdit. The signal peptide was predicted by both neural networks and hidden Markov models on a Signal IP 3.0 Server [41]. The isoelectric point (pI) and molecular weight of the deduced protein were determined by “Compute pI/MW” tool on the ExPASY Server (http://www.expasy.org/tools) [42]. The sequence similarity search was conducted using BLAST at the National Center for Biotechnology Information (http://www.ncbi.nlm.nih.gov/blast). Phylogenetic analysis was conducted with amino acid sequences using the program Mega 3.0 [43]. The phylogenetic tree was constructed by the method of Yue et al (2011) [44].

### Immunofluorescence Analysis of Laminin

Mantle tissues of the clam, *M. meretrix*, were dissected and fixed with 4% paraformaldehyde for 24 h. The fixed tissues were embedded in paraffin, cut into slices 7 mm thick, and then transferred to poly-L-lysine-coated slides (Boster, Wuhan, China). Immunofluorescence examination was conducted by referring to the “immunofluorescence” staining protocol on the website IHCWORLD (http://www.ihcworld.com/_protocols/general_IHC/immunofl.htm). Rat anti-laminin monoclonal antibody (Cwbiotech, Beijing, China) was used as the primary antibody at a dilution of 1∶50. The secondary antibody was goat anti-rat IgG-FITC (Solarbio, Beijing, China) at a dilution of 1∶50. The negative staining control procedure was performed without adding primary antibody. Fluorescence signals were visualized with a confocal microscopy system (Carl Zeiss International, Germany).

### Expression of Recombinant MmeLR (rMmeLR) Protein and Preparation of Anti-rMmeLR Polyclonal Antibodies

A DNA fragment containing the predicted MmeLR coding region plus a BamH I restriction site at the 5′ end and an EcoR I restriction site at the 3′ end was generated by PCR using the primers LRBamH I-F and LREcoR I-R (Table 2). After BamH I and EcoR I cleavage, the DNA fragment was inserted into a pGEX-4T-1 plasmid (GE Healthcare, USA) containing an N-terminal GST affinity tag to construct a pGEX-MmeLR recombinant plasmid. The expression of the GST-MmeLR fusion protein (rMmeLR) was induced following the method of Yue et al (2011), and the rMmeLR was denatured and refolded by stepwise dialysis according to the protocol of Yue et al (2011) [44].

The refolded rMmeLR was then purified by affinity chromatography using a GSTrap FF column (GE Healthcare, USA), following the manufacturer’s protocol. Purified rMmeLR was identified by SDS-PAGE in 12% gels with Coomassie brilliant blue R250 (CBB-R250) staining. The purified rMmeLR protein was sent to the Hangzhou HuaAn Biotechnology Company (Hangzhou, China) to prepare the anti-rMmeLR polyclonal antibodies. Western blotting assays were conducted to test the specificity of the polyclonal antibodies for rMmeLR.

### Quantitative Analysis of MmeLR Expression by Realtime PCR

MmeLR mRNA expression in different tissues of *M. meretrix* was analyzed by realtime PCR. The β-actin gene from *M. meretrix* was chosen as the endogenous reference gene using the primers w-act-f and w-act-r (Table 2). The MmeLR specific primers were rtLR-f and rtLR-r. All primers were optimized by temperature gradients for the determination of the proper annealing temperature. In addition, blank controls with no templates were tested, and melting curves were analyzed to check the quality of the primers before the experiments. The cDNAs from different tissues were used as templates. The amplification was carried out in triplicate on an ABI PRISM 7000™ Thermocycler (ABI, USA), following the formula of Yue et al (2011) [44]. The amplification was carried out in 25 µL reactions containing 1 µL samples of cDNA, 5 pmol of each primer, 0.2 mmol of dNTP, 0.25 µL Blend Taq Plus (ToYoBo, Japan) with 2.5 µL Blend Taq buffer, 1.25 µL Eva Green (20× in water, ToYoBo, Japan), and 5% DMSO (v/v). The program for realtime PCR of the β-actin gene was 1 cycle of 94°C for 2 min and 40 cycles of 94°C for 15 s, 53°C for 15 s, and 72°C for 20 s. The program for realtime PCR of the MmeLR gene was 1 cycle of 94°C for 2 min and 40 cycles of 94°C for 20 s, 56°C for 20 s, and 72°C for 20 s.

### Detection of MmeLR Protein using Western Blots

Total protein was extracted using a Total Protein Extraction Kit (BestBio, Shanghai, China) according to the manufacturer’s protocol. Protein concentrations were determined by the Bradford protein assay (Bio-Rad, USA) standardized with a BSA concentration gradient. Equal quantities of total protein from each sample were separated in 12% SDS-polyacrylamide gels and transferred onto a PVDF membrane by electroblotting at 100 V for 1 h. The membranes were blocked at 4°C overnight in 5% skimmed milk and then incubated with rabbit anti-β-actin IgG (Cwbiotech, Beijing, China) and rabbit anti-rMmeLR IgG in Tris buffered saline, pH 8.0 (TBS) containing 0.05% skimmed milk at 37°C for 1 h, respectively. The incubated membranes were washed three times (10 min each) with TBS, and then incubated with horseradish peroxidase-conjugated goat anti-rabbit IgG solution (Huabio, Hangzhou, China) in TBS containing 0.05% skimmed milk powder at 37°C for 1 h. After the membranes were washed three times (10 min each) in TBS, the bands on the membranes were visualized using a HRP-DAB assay kit (Tiangen, Beijing, China).

### Far-western Analysis

To confirm the interaction between laminin and rMmeLR, far-western blotting was performed as described by Wan et al (2008) with some modifications [45]. In a previous study, we produced a recombinant caspase (rCaspase) from oysters, which was used as a negative control in the present research. Laminin (1 mg/ml, natural mouse, Invitrogen, USA) or rCaspase (1 mg/ml) was directly blotted onto PVDF membranes, respectively. Subsequently, the membranes containing laminin or rCaspase were incubated with purified rMmeLR (3 µg/ml) or GST protein (3 µg/ml, the negative control) in renaturation buffer for 2 h at RT, followed by incubation with anti-MmeLR polyclonal antibodies (1∶10,000) for 2 h at RT. Then, western blotting assays were conducted as described above.

### Primary Cell Culture and Binding Assays of MmeLR and Laminin

After acclimatization, the clams were placed into aerated sterile seawater for 6 h before dissection. The mantle tissues were isolated and then washed for 20 min six times in sterile seawater containing penicillin 100 U/ml, streptomycin 100 µg/ml and gentamycin 100 µg/ml. Tissues were washed again in sterile modified L15 medium (Gibco, USA) with 1 g/L glucose, 2.4 g/L HEPES, 12 g/L NaCl, 1.2 g/L NaHCO_3_, 5 g/L yeast extract and the three antibiotics mentioned above for 30 min. The washed tissues were cut into fragments, mixed with fresh medium, and centrifuged three times at 1200 rpm for 5 min. Cells were cultured in 25 cm^2^ cell culture flasks (Corning, USA) in an incubator (SANYO, Japan) at 26°C for 24 h before the binding experiment.

The primary cells used for binding assays were collected in 24-well culture clusters (Corning, USA). The plates of the matrigel-coated group were coated with matrigel (BD, USA), which contains laminin as its main component [9], while the plates of the control group were free of matrigel. The cells of the matrigel-coated group and the control group were harvested after being cultured for 0 h, 12 h, 24 h, 48 h, 72 h, 6 days and 12 days. Samples at each time point were collected and treated either by quick freezing in liquid nitrogen for RNA extraction or by immediately extracting protein for western blot analysis. There were three replicates for each group.

The primary cells for the laminin antibody analysis were cultured as described above. The 24 well cell culture clusters were coated with different concentrations (0, 3, 30, or 60 µg/ml) of laminin at 4°C overnight. The coated clusters were then incubated with 20 µg/ml rat anti-laminin monoclonal antibody (Cwbiotech, Beijing, China) at 37°C for 1 hour to saturate the laminin. The cells were plated and cultured at 26°C for 48 h, harvested, and directly frozen in liquid nitrogen for RNA extraction. There were three replicates for each group.

The bcl-2 and p53 genes were used as indicator genes for the detection of cell apoptosis. Realtime PCR was carried out to analyze the relative expression of bcl-2 and p53 mRNA using the primers bcl-2f, bcl-2r, p53f, and p53r (Table 2), and a program of 1 cycle of 94°C for 2 min, 40 cycles of 94°C for 20 s, 55°C for 20 s, and 72°C for 40 s.

### Statistical Analysis

The differences in Ct values for the reference β-actin and the target gene were the basis for the relative expression analysis for each sample. The relative gene expression data were analyzed using the 2^−ΔΔCT^ method [46]. Data were examined for homogeneity of variances (Levene’s test) and then analyzed by One-way ANOVA using Tukey’s test. A *P*<0.05 was considered significant. All statistical analyses were conducted with a statistical package (SPSS 16.0 for Windows).
